# Effect of long-term cannabidiol on learning and anxiety in a female Alzheimer’s disease mouse model

**DOI:** 10.3389/fphar.2022.931384

**Published:** 2022-09-27

**Authors:** Rose Chesworth, David Cheng, Chloe Staub, Tim Karl

**Affiliations:** ^1^ School of Medicine, Western Sydney University, Campbelltown, NSW, Australia; ^2^ Neuroscience Research Australia, Randwick, NSW, Australia

**Keywords:** Alzheimer’s disease, behaviour, cannabidiol (CBD), spatial memory, female, amyloid precursor protein, presenilin 1

## Abstract

Cannabidiol is a promising potential therapeutic for neurodegenerative diseases, including Alzheimer’s disease (AD). Our laboratory has shown that oral CBD treatment prevents cognitive impairment in a male genetic mouse model of AD, the *amyloid precursor protein 1 x presenilin 1* hemizygous (*APPxPS1*) mouse. However, as sex differences are evident in clinical populations and in AD mouse models, we tested the preventive potential of CBD therapy in female *APPxPS1* mice. In this study, 2.5-month-old female wildtype-like (WT) and *APPxPS1* mice were fed 20 mg/kg CBD or a vehicle *via* gel pellets daily for 8 months and tested at 10.5 months in behavioural paradigms relevant to cognition (fear conditioning, FC; cheeseboard, CB; and novel object recognition test, NORT) and anxiety-like behaviours (elevated plus maze, EPM). In the CB, CBD reduced latencies to find a food reward in *APPxPS1* mice, compared to vehicle-treated *APPxPS1* controls, and this treatment effect was not evident in WT mice. In addition, CBD also increased speed early in the acquisition of the CB task in *APPxPS1* mice. In the EPM, CBD increased locomotion in *APPxPS1* mice but not in WT mice, with no effects of CBD on anxiety-like behaviour. CBD had limited effects on the expression of fear memory. These results indicate preventive CBD treatment can have a moderate spatial learning-enhancing effect in a female amyloid-β-based AD mouse model. This suggests CBD may have some preventive therapeutic potential in female familial AD patients.

## 1 Introduction

Recently, there has been increasing interest in cannabidiol (CBD), a non-intoxicating phytocannabinoid compound in the *Cannabis sativa L.* [Cannabaceae] plant, for the treatment of several neurodegenerative and psychiatric disorders. CBD possesses antioxidant, anti-apoptotic, neuroprotective, and anti-inflammatory properties [reviews: (Scuderi et al., 2009; Campos et al., 2016)]. This is particularly relevant for brain disorders characterised by neuroinflammation and cell death including neurodegenerative disorders such as Alzheimer’s disease (AD), which has no cure. Dementia affects over 55 million people globally, of which AD is the most common form (Wimo et al., 2015). AD is characterised by the presence of extracellular amyloid-beta (Aβ) plaques and intracellular neurofibrillary tangles consisting of hyperphosphorylated tau (Bloom, 2014); these are found in the neocortex (Aβ) and the transentorhinal cortex (tau) in early disease stages but spread throughout the brain as the disease progresses (Braak and Braak, 1991; Thal et al., 2002). Inflammatory markers [e.g., interleukin (IL)-1, IL-6, tumour necrosis factor (TNF)-α, and activated microglia] and markers for oxidative stress [e.g. oxidised proteins and oxidative modifications in nuclear and mitochondrial DNA (Gella and Durany, 2009; Chen and Zhong, 2014)] are also commonly found in AD postmortem brain tissue (McGeer et al., 2016) and are hypothesised to precede the development of Aβ and tau pathology (Holmes, 2013). Targeting inflammation is of increasing interest as an AD treatment approach (McGeer et al., 2016). The failure of anti-inflammatory therapies to date may be due to missing the therapeutic window (Rivers-Auty et al., 2020) or requiring multimodal drug strategies to target a complex disease (Karl et al., 2017). Considering the anti-inflammatory, anti-apoptotic, and neuroprotective properties of CBD, there is growing interest in its potential for the treatment of AD (Karl et al., 2017).

*In vitro* data indicate CBD can reduce AD-relevant pathology [reviews: (Karl et al., 2017; Watt and Karl, 2017)]. CBD inhibits tau hyperphosphorylation (Esposito et al., 2006a; Vallee et al., 2017), reduces full-length APP expression, and reduces Aβ peptide expression (Scuderi et al., 2014), suggesting CBD can reduce AD pathology in cell culture. CBD also improves cell survival and reduces the production of reactive oxygen species and nitric oxide production (Iuvone et al., 2004; Esposito et al., 2006b; Amini and Abdolmaleki, 2022), suggesting CBD can reduce Aβ-induced toxicity. CBD can also protect against cell viability loss induced by Aβ_42_ (Janefjord et al., 2014), which is a major component of amyloid plaques (Gu and Guo, 2013). CBD reduces microglial function and cytokine gene and protein expression after intracerebroventricular (i.c.v.) or hippocampal Aβ administration to mice (Esposito et al., 2007; Martin-Moreno et al., 2011) and can upregulate the immune system function and increase autophagy in AD models (Hao and Feng, 2021), which may be another mechanism by which CBD improves AD pathology. CBD may also have therapeutic effects in AD by acting on hippocampal long-term potentiation (LTP); pretreatment with CBD prevents the Aβ_1-42_ oligomer-induced reduction in hippocampal CA1 LTP in mice (Hughes and Herron, 2019), thereby reversing effects of AD pathology on synaptic plasticity.

Preclinical *in vivo* data suggest remedial CBD treatment *via* i. p. administration reverses cognitive impairment in pharmacological and genetic mouse models for Alzheimer’s disease [reviews: (Karl et al., 2017; Watt and Karl, 2017)]. For example, chronic CBD prevents learning and memory impairments in mice injected with Aβ intraventricularly (Martin-Moreno et al., 2011). Also, in a mouse model of familial AD (Cheng et al., 2014a; Aso et al., 2015; Coles et al., 2020; Watt et al., 2020a), i.e., mice hemizygous for *amyloid precursor protein* (*APP*) and *presenilin 1* (*PS1*) genes (i.e. *APPxPS1* mice), they are characterised by increased Aβ accumulation and accelerated plaque pathology from 4 months of age (Wang et al., 2003) and spatial learning and memory deficits from 7 to 8 months of age (Cao et al., 2007; Reiserer et al., 2007). Therapeutic effects of CBD in APPxPS1 mice have been found at different CBD doses [range of 5–50 mg/kg (Cheng et al., 2014a; Coles et al., 2020; Watt et al., 2020a)] and also when using CBD-enriched extracts (Aso et al., 2015). The mechanisms involved are not entirely clear. Chronic CBD has moderate effects on Aβ levels in the hippocampus (Watt et al., 2020a) and reduces the astrocytic response and cell surface adhesion molecule CCL4 mRNA expression in *APPxPS1* mice (Aso et al., 2015). However, to date, remedial CBD treatment has not been shown to strongly affect other AD-relevant receptors and molecules in *APPxPS1* mice, including brain-derived neurotrophic factor (BDNF), proliferator-activated receptor γ (PPARγ), ionised calcium-binding adaptor molecule 1 (IBA1) and various cytokines (Watt et al., 2020a).

In addition to the remedial effects (i.e., CBD administered when behavioural impairment is present), CBD has been found to prevent the development of AD-relevant behavioural impairments. When CBD is administered orally for 8 months from 2.5 months of age, CBD prevents the development of social recognition impairment in male *APPxPS1* mice (Cheng et al., 2014c). In this study, there were also subtle effects of CBD on neuroinflammation and cholesterol in the cortex and dietary phytosterol retention in the cortex and hippocampus (Cheng et al., 2014c). This suggests CBD has potential preventive and pro-cognitive effects on AD in male animals.

Despite this, the potential preventive effects of CBD treatment on cognition in female *APPxPS1* mice are unknown. This is a critical question as sex differences are evident in AD: there is a higher prevalence of AD in women, and women suffer greater cognitive deterioration than men at the same disease stage (Laws et al., 2018; Medeiros and Silva, 2019). Importantly, sex differences are also found in the *APPxPS1* mouse model, e.g., social novelty recognition impairment is evident in male *APPxPS1* mice but not in female mice, while spatial memory impairment is evident in female *APPxPS1* mice but not in male *APPxPS1* mice (Cheng et al., 2013; Cheng et al., 2014b). Female *APPxPS1* mice also show greater amyloid burden and higher plaque number (Wang et al., 2003), as well as higher levels of phosphorylated tau and proinflammatory cytokines, more severe astrocytosis and microgliosis, and greater neuronal and synaptic degeneration than male mice at the same age (Jiao et al., 2016). These sex differences make the *APPxPS1* mice an appropriate model to investigate potential sex differences in CBD’s efficacy for treating cognitive impairment in AD. Furthermore, remedial CBD treatment (i.e., after the development of cognitive deficits) affects different domains in male and female *APPxPS1* mice: CBD improves social recognition, object recognition, and spatial reversal learning in male *APPxPS1* mice (Cheng et al., 2014a; Watt et al., 2020a) but only object recognition deficits in female *APPxPS1* mice (Coles et al., 2020). Indeed, there has been limited investigation of sex differences in CBD’s effects on anxiety-like behaviour and cognition, e.g., (Osborne et al., 2017; Osborne et al., 2019; Garcia-Baos et al., 2021), highlighting the importance of examining female and male animals. Thus, we sought to determine if *preventive* CBD affects different behavioural domains in male and female *APPxPS1* mice. Finally, we assessed a preventative approach because treatment after symptom onset may be too late to limit ongoing neurodegenerative processes in AD (Lee et al., 2022), and thus, treatments with preventative potential could have significant clinical impact by limiting disease progression and symptom onset.

Thus, the present study was designed to complement earlier behavioural research in our laboratory (Cheng et al., 2014c), to determine if 20 mg/kg CBD treatment given orally *via* gel pellets for 8 months prevents the development of the AD-relevant behavioural phenotype in *APPxPS1* female mice.

## 2 Materials and methods

### 2.1 Animals

*APPxPS1* hemizygous mice on a congenic C57BL/6JxC3H/HeJ background were generated, as described previously (Cheng et al., 2013; Cheng et al., 2014a; Cheng et al., 2014b; Cheng et al., 2014c). These mice were originally described by Borchelt et al. (1997). They express the “humanized” mouse amyloid beta precursor protein gene modified at three amino acids to reflect the human residues and further modified to contain the K595N/M596L (also called K670N/M671L) mutations linked to familial Alzheimer’s. They also express a mutant human presenilin 1 carrying the exon-9-deleted variant (PSEN1dE9) associated with familial Alzheimer’s disease. These gene mutations are controlled by mouse prion protein promoter elements, directing transgene expression predominantly to CNS neurons.

Mice were bred at Australian BioResources (ABR: Moss Vale, NSW, Australia), where they were group housed in individually ventilated cages (Type Mouse Version 1: Airlaw, Smithfield, Australia) under a 12/12 h light/dark cycle with a dawn/dusk simulation. Mice were transported to the Neuroscience Research Australia animal facility (Randwick, Australia) at ∼10 weeks of age, where littermates were group housed (two to three mice per cage) in polysulfone cages (1144B: Techniplast, Rydalmere, Australia) with corn cob bedding (PuraCob Premium: Able Scientific, Perth, Australia) and tissues for nesting. Mice were kept under a 12:12 h light:dark schedule [light phase: white light (illumination: 210 lx); lights on 0700–1900 h]. Environmental temperature was automatically regulated at 21 ± 1°C, and relative humidity was 40–60%. Food (Gordon’s Rat and Mouse Maintenance Pellets: Gordon’s Specialty Stockfeeds, Yanderra, Australia) and water were provided *ad libitum*, except where specified.

Research and animal care procedures were approved by the University of New South Wales Animal Care and Ethics Committee in accordance with the Australian Code of Practice for the Care and Use of Animals for Scientific Purposes. *APPxPS1* mice and their non-transgenic wild type-like littermates (WT) were approximately 2.4 months of age at the onset of the study. The number of animals per group was as follows: 14 WT-vehicle, 16 *APPxPS1*-vehicle, 14 WT-CBD, and 12 *APPxPS1*-CBD.

### 2.2 Drugs

Powdered CBD (CAS: 13956-29-1, THC Pharm GmbH, Frankfurt/Main, Germany) was used at a dose of 20 mg/kg body weight, based on previous work in our laboratory (Cheng et al., 2014a; Cheng et al., 2014c). CBD was administered in gel pellets to prevent the stress of chronic injections on behavioural and cognitive results; methods were identical to those published previously (Cheng et al., 2014c). Briefly, CBD or the vehicle were dissolved in a highly palatable, sweetened, and chocolate-flavoured gel pellet and administered at a volume of 8 ml/kg body weight. CBD was dissolved in gel pellets with a final composition of 2.0% ethanol, 2.0% Tween 80, 15.2% Splenda (Splenda Low Calorie Sweetener: Johnson & Johnson Pacific Pty, Broadway, Australia), 8.7% gelatine (Davis Gelatine: GELITA Australia Pty, Josephville, Australia), 20.1% chocolate flavouring (Queen Flavouring Essence Imitation Chocolate: Queen Fine Foods Pty, Alderley, Australia), and 52.0% water for irrigation. Vehicle gel pellets were identical but contained no CBD.

### 2.3 Treatment schedule

Mice were initially habituated to vehicle gel pellets in their home cages for 7 days prior to the start of treatment. Following this, mice were isolated within their home cages during treatment by placing a plastic divider in the home cage. Then, animals were given either a vehicle or a CBD gel pellet (treatments were quasi-randomized), which they consumed within 2–5 min. Mice did not need to be food-deprived to ensure they ate the gel pellet. A trained experimenter watched all the animals consume the gel pellets daily to ensure the correct dose was administered each day. The plastic divider was removed once the mice had consumed the gel pellets. Mice were treated daily for 8 months (i.e., from 2.5 to 10.5 months of age) late in the afternoon, to avoid potential effects of acute CBD on test outcomes.

### 2.4 Behavioural testing

Starting at 10 months of age, mice were tested with an inter-test interval of at least 48 h (Cheng et al., 2014c). We chose paradigms based on the baseline behavioural phenotype previously reported in these mice in our laboratory (Cheng et al., 2014b). This strategy was chosen rather than directly replicating the test biography of CBD-treated *APPxPS1* male mice (Cheng et al., 2014c)] as female AD transgenic mice exhibit a different cognitive profile to males, i.e., only females exhibit impaired spatial memory (Cheng et al., 2014b), whereas only transgenic males show impaired social recognition memory (Cheng et al., 2013). All tests were conducted during the first 5 h of the light phase to minimize the effects of the circadian rhythm. All test apparatus was cleaned with 70% v/v ethanol in between test animals. Behavioural tests were conducted in the following order: fear conditioning, cheeseboard, elevated plus maze, and novel object recognition.

#### 2.4.1 Fear conditioning (FC)

FC assesses hippocampal- and amygdala-dependent associative learning, and methods were identical to those published previously (Cheng et al., 2013; Cheng et al., 2014a; Cheng et al., 2014c). During conditioning, mice were placed into the test chamber (Model H10-11R-TC, Coulbourn Instruments, United States) for 2 min. An 80 dB conditioned stimulus (CS) was presented twice for 30 s with a co-terminating 0.4-mA 2-s foot shock (unconditioned stimulus; US) with an inter-pairing interval of 2 min. The test concluded 2 min later. The next day (context test), mice were returned to the apparatus for 7 min. On day 3 (cue test), animals were placed in an altered context for 9 min. After 2 min (pre-CS/baseline), the CS was presented continuously for 5 min. The test concluded after another 2 min, without the CS. Time spent *freezing* was measured by Any-Maze^TM^ software.

#### 2.4.2 Cheeseboard (CB)

Spatial memory was assessed in the CB using established methods (Cheng et al., 2014b; Coles et al., 2020; Watt et al., 2020a). Sweetened condensed milk, 1:4 in water, was used as a food reward, and mice were food-restricted during CB training and testing (access to food for 1–2 h, following completion of daily testing, mice kept at 85-90% of free feeding body weight). There were three trials per day, except at the probe, where there was one trial. All trials were 2 min, unless the food reward was located in <2 min, with a 20-min intertrial interval (ITI).

Mice were habituated to the blank side of the board for 2 days. Next, mice were trained for 7 days to locate a well containing a food reward. The latency of the mice to find the target well was recorded, and if the food reward was not located within 2 min, the mouse was gently guided to the well by the experimenter. Mice were considered to have learnt the task if the average latency of all three trials in 1 day was <20 s. After 7 days, our control group (WT VEH) met acquisition criteria. The next day, a probe trial was conducted to assess spatial reference memory. No wells were baited, and mice were given 2 min to explore the apparatus freely. To assess if animals could update their spatial learning contingencies, we conducted reversal learning, whereby the location of the food reward was changed. Mice completed 4 days of reversal training before the reversal probe trial (WT VEH mice met reversal criteria in 4 days), which was conducted 24 h after reversal training. During the reversal probe, no wells were baited and mice were given 2 min to explore the apparatus freely. Mice were returned to free feeding, following completion of the CB, and subsequent behavioural tests were conducted, and only once mice had returned to free feeding weight.

The average latency to find the reward was analysed as a general indication of learning, and this was used to determine when mice acquired the task (Cheng et al., 2014b; Coles et al., 2020). The first trial per day across training was also analysed to assess long-term reference memory (retention of ≥24 h), and the average of trials 2 and 3 each day across training was analysed to assess intermediate-term memory [retention falling between short-term (<2 min) and long-term (>24 h) memory] (Taglialatela et al., 2009; Coles et al., 2020). The average speed and distance were analysed throughout acquisition and reversal learning. At probe tests, the time spent in the different CB zones (i.e., board was separated into 8 equal zones, corresponding with the lines of wells) and the average speed and distance travelled were measured by Any-Maze^TM^ software.

#### 2.4.3 Elevated plus maze (EPM)

The EPM assesses the natural conflict between the tendency of mice to explore a novel environment and their avoidance of a brightly lit, elevated, and open area (Montgomery, 1955). Methods have been described previously (Cheng et al., 2013; Cheng et al., 2014b). The ‘+’ apparatus consisted of two alternate open arms (35 cm × 6 cm; without side walls) and two alternate enclosed arms (35 cm × 6 cm; height of enclosing walls 28 cm) connected by a central platform (6 cm × 6 cm), elevated 70 cm above the floor. Mice were placed at the centre of the ‘+’ of the grey PVC plus maze, facing an enclosed arm, and were allowed to explore the maze for 5 min. The time spent on open arms, entries into the open arms, and the distance travelled on the open and enclosed arms were recorded by AnyMaze™ tracking software.

#### 2.4.4 Novel object recognition test

The innate preference of a mouse for novelty and its ability to distinguish a novel object from a familiar object (Dere et al., 2007) are utilised in the NORT. The NORT was conducted over 3 days [methods: (Cheng et al., 2014a)]. Two 10-min trials were conducted per day, with a 1 h ITI. On day 1, mice were habituated to the empty arena during both trials. On day 2, mice were habituated to the empty arena during trial 1 and to two identical objects during trial 2. On the test day (day 3), mice were exposed to two identical objects in the training trial (objects distinct from day 2) and then one familiar and one novel object in the test trial. The objects used were a mini Rubik’s cube and a plastic garden hose nozzle. The objects and their locations were counterbalanced across genotypes and treatment groups. Time spent *nosing* and *rearing* on the objects was recorded by AnyMaze™ tracking software and confirmed by manual scoring. The percentage of time spent *nosing* the novel object indicated short-term object recognition memory (% novel object recognition) and was calculated using [(novel object *nosing* time/novel + familiar object *nosing* time) × 100]. The percentage of time spent *nosing* and *rearing* was combined to create an “*exploration”* score, and the percentage of novel object *exploration* was calculated in the same way as % *nosing*.

### 2.5 Statistical analysis

Data were analysed using SPSS Statistics 25 (IBM, NY, United States). Three- and two-way repeated measures (RM) analysis of variance (ANOVA) with within factors “minutes” (FC) or “cue” (FC) and between factors “genotype” (WT vs. *APPxPS1*) and “treatment” (VEH vs. CBD 20 mg/kg) was conducted. Where interactions were found, we conducted subsequent two- and one-way ANOVA split by the corresponding factor, as published previously (Long et al., 2012; Cheng et al., 2014a; Cheng et al., 2014c; Coles et al., 2020; Watt et al., 2020a). *Post hoc* effects are shown in figures only. Data from fear conditioning and cheeseboard were analysed with three-way ANOVA but are presented in separate graphs for visual clarity.

Data for the FC cue test were also analysed as total *freezing* in the 2 min prior to tone presentation, the 5 min during tone presentation, and the 2 min post-tone. Data for NORT, CB probe, and CB reversal probe tests were analysed using single-sample t-tests comparing data to the chance level for each test (Cheng et al., 2013; Cheng et al., 2014b; Coles et al., 2020). The chance level for NORT is 50% (1/2 objects), and for CB, it is 12.5% (1/8 zones). Data were presented as mean ± SEMs, and differences were regarded as statistically significant if *p* < 0.05.

Exclusions: FC: one WT CBD-treated mouse was excluded due to high baseline *freezing* (>2.5 SDs above the mean for that group). CB: three mice (1x WT VEH, 2x *APPxPS1* CBD) were excluded from the CB analysis as their latency to find the food reward did not decrease across days (i.e., stayed at 120 s for the 7 days of training), so they did not engage with the paradigm.

## 3 Results

### 3.1 Fear conditioning

**FIGURE 1:**
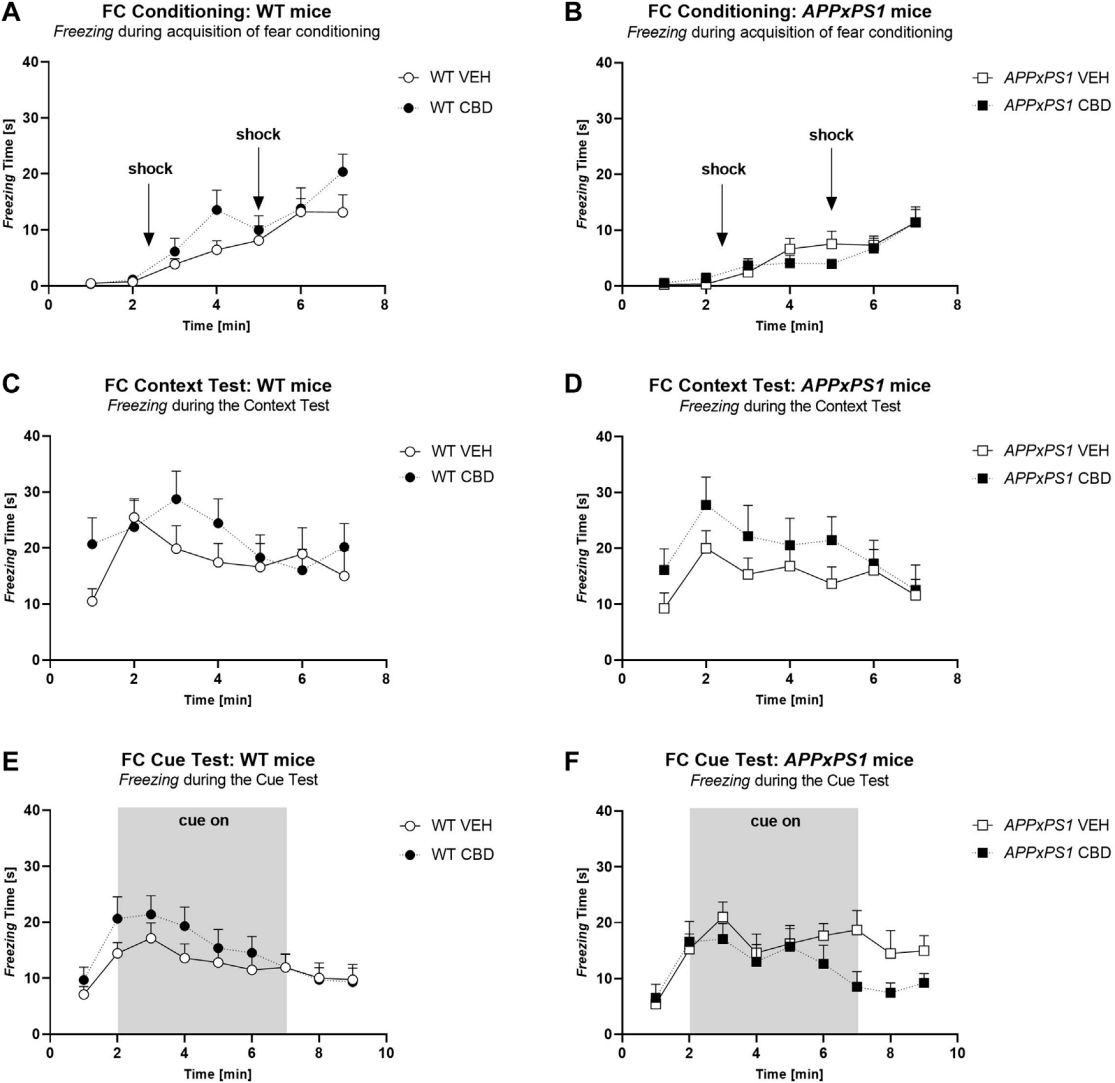
*Freezing* time [s] during **(A,B)** acquisition of fear conditioning, **(C,D)** context test, and **(E,F)** cue test in *APPxPS1* and WT female mice treated daily with 20 mg/kg CBD or VEH for 8 months. Interactions were present in (A, B) between “minutes” × “genotype” (*p* = 0.02) and in **(E,F)** between “minutes” × “treatment” (*p* = 0.02). Data were analysed using three-way RM ANOVA and presented as mean ± SEM in separate graphs for visual clarity. N = 14 WT VEH, 16 *APPxPS1* VEH, 14 WT CBD, and 13 *APPxPS1* CBD. Abbreviations: *APPxPS1*: *amyloid precursor protein x presenilin 1*; CBD: cannabidiol; VEH: vehicle; WT: wildtype-like.

There were no “genotype” or “treatment” differences in baseline *freezing* during conditioning (i.e., the first 2 min of the test), indicating baseline genotype or treatment differences did not confound the interpretation of subsequent analyses (all “treatment” and “genotype” *p*-values > 0.05; Table 1). During acquisition of fear conditioning, all mice increased their *freezing* behaviour as the test progressed, indicating acquisition of the tone-shock association [“minutes” F (6,306) = 40.3, *p* < 0.0001]. Although there was no overall effect of “treatment” on *freezing* [F (1,52) = 1.0, *p* = 0.3; no “treatment” interactions, all *p*-values > 0.05], a “minutes” by “genotype” interaction was detected [F (6,306) = 2.5, *p* = 0.02]. However, when split by “genotype”, both genotypes increased their *freezing* as the test progressed, irrespective of CBD treatment (all “time” *p*-values < 0.0001, no main “treatment” main effects, or interactions with ‘treatment’) (Figures 1A,B).

**TABLE 1:** Freezing during fear conditioning. Percentage of *freezing* within each time block [%] during the first 2 min on conditioning day and during the cue test.

Measure	WT VEH	WT CBD	*APPxPS1* VEH	*APPxPS1* CBD
Baseline *freezing* (first 2 min of conditioning)	1.00 ± 0.42	1.25 ± 0.50	0.58 ± 0.25	1.83 ± 1.08
Cue test: *freezing* pre-cue	17.92 ± 2.33	25.25 ± 4.92	17.25 ± 3.08	19.25 ± 4.83
Cue test: *freezing* during cue	22.37 ± 3.87	27.53 ± 4.13	29.40 ± 4.13	22.30 ± 3.73
Cue test: *freezing* post-cue	16.5 ± 4.00	24.58 ± 4.75	24.58 ± 4.75	13.92 ± 2.42

In the context test, there were no effects of “genotype” [F (1,51) = 0.7, *p* = 0.4] or “treatment” [F (1,51) = 2.3, *p* = 0.1] on *freezing* in the shock-associated environment, and no interactions were detected (all *p*-values > 0.05) (Figures 1C,D). All mice, regardless of treatment or genotype, showed higher levels of *freezing* earlier in the test, which decreased as the test progressed [“minutes” F (6,306) = 6.8, *p* < 0.0001; no interactions] (Figures 1C,D).

During the cue test, there were no overall effects of “genotype” [F (1,51) = 0.1, *p* = 0.9] or “treatment” [F (1,51) = 0.1, *p* = 0.8]. There was an interaction between “minutes” × “treatment” [F (8,408) = 2.2, *p* = 0.02], suggesting CBD-treated animals *froze* less than VEH-treated animals, particularly in the 2^nd^ half of the test, although follow-up analyses splitting by corresponding factors revealed no further significant differences (all *p*-values > 0.1) (Figures 1E,F). When data were analysed according to total time spent *freezing* pre-cue, during cue presentation, and post-cue, there were no effects of “genotype” or “treatment” and no interactions (all *p*-values > 0.05, Table 2).

**TABLE 2:** Open arm measures in the elevated plus maze test. Open arm entries [n] and the open arm distance ratio [%] in WT and *APPxPS1* mice, following chronic treatment with a vehicle or 20 mg/kg CBD. Data presented as mean ± SEM.

Measure	WT VEH	WT CBD	*APPxPS1* VEH	*APPxPS1* CBD
Open arm entries [n]	4.57 ± 1.48	3.93 ± 1.19	2.47 ± 0.67	2.08 ± 0.79
Open arm distance ratio [%]	5.25 ± 2.02	9.73 ± 3.1	8 ± 4.31	5.66 ± 3.15

### 3.2 Cheeseboard

#### 3.2.1 Acquisition

**FIGURE 2:**
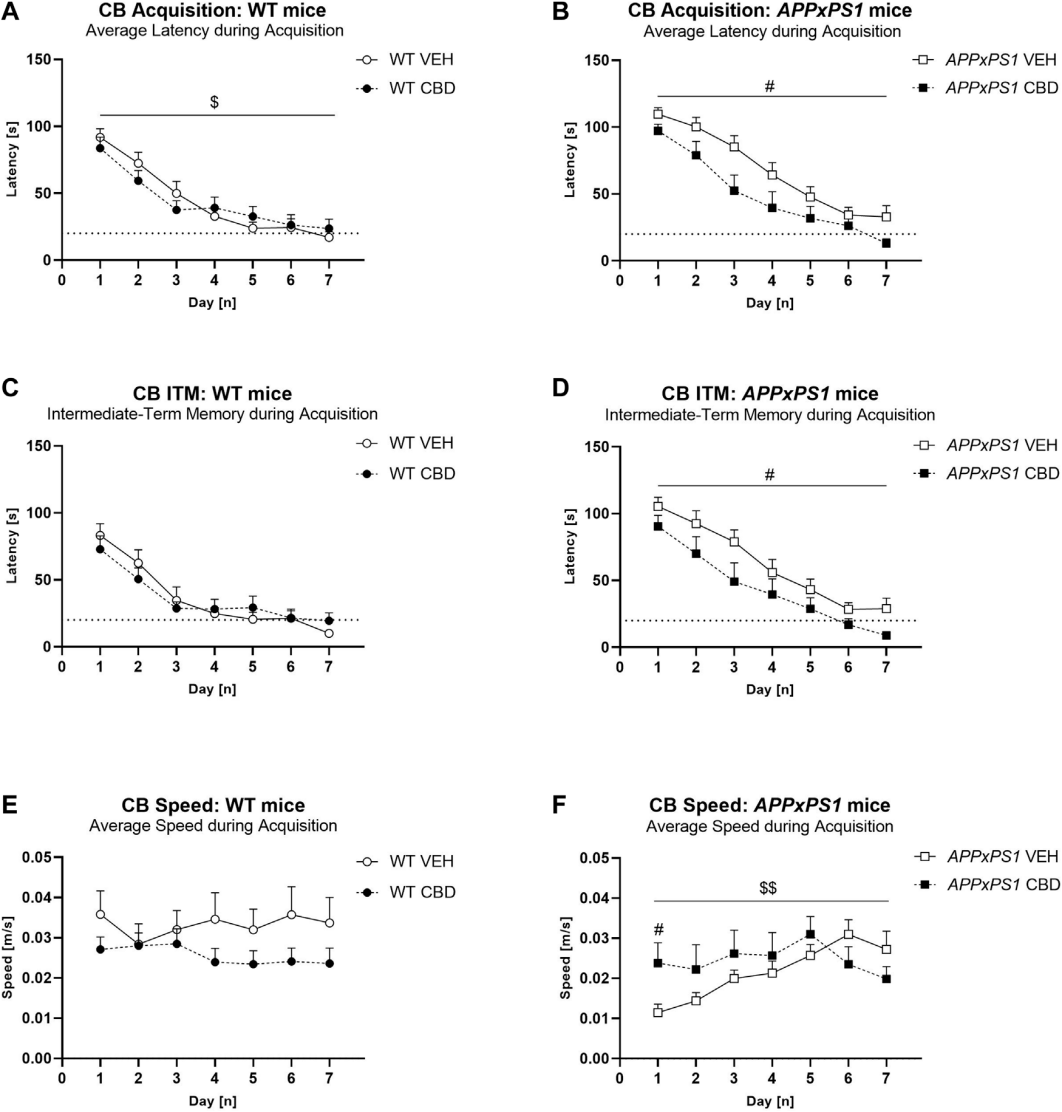
Long-term CBD improves average latency, intermediate-term memory latency, and speed during cheeseboard acquisition in APPxPS1 female mice. Latency [s] and speed [m/s] when finding the food reward in the cheeseboard in *APPxPS1* and WT mice treated daily with 20 mg/kg CBD for 8 months. **(A,B)** Average latency [s] to find the food reward (averaged across 3 trials per day) during acquisition of the cheeseboard task. **(C,D)** Intermediate-term memory latency [s] (i.e., average latency for trials 2 and 3 of each day) during acquisition. **(E,F)** Average speed [m/s] (averaged across three trials per day) during cheeseboard acquisition. The dotted line in **(A–D)** indicates the 20-s cut-off threshold for acquisition. In **(A,B),** a “days” × “genotype” interaction (*p* = 0.02) was detected, and in **(C,D),** a “days“ × “genotype” interaction (*p* = 0.004) was detected. In (**E,F)**, a “days“ × “treatment” interaction was detected; when split by “treatment,” there was a “days” × “genotype” interaction (*p* = 0.02) in VEH-treated mice. Splitting by “day” confirmed “genotype” differences on days 1–3 (*p*-values < 0.02). Data analysed using three-way RM ANOVA and presented as mean ± SEM in separate graphs for visual clarity. When data were split by the corresponding factor, significant “treatment” effects in *APPxPS1* mice are indicated by hash symbols (^#^ *p* < 0.05); interactions between “treatment” and “days” are indicated by ‘$’ (^$^ *p* < 0.05; ^$$^ *p* < 0.01). N = 13 WT VEH, 16 *APPxPS1* VEH, 14 WT CBD, and 10 *APPxPS1* CBD. Abbreviations: *APPxPS1*: *amyloid precursor protein x presenilin 1*; CB: cheeseboard; CBD: cannabidiol; ITM: intermediate-term memory; VEH: vehicle; WT: wildtype-like.

Averaging latency to find the food reward from all three trials on each day, we found that all experimental groups reduced their latency during acquisition, indicating they learnt the location of the food reward [“days” F (6,294) = 102.1, *p* < 0.0001]. Generally, *APPxPS1* mice had longer latencies than WT mice [“genotype” F (1,49) = 5.7, *p* = 0.02]. The latency improved across days to match control levels by the last 2 days of training [“days” × “genotype” F (6,294) = 2.5, *p* = 0.02]. CBD treatment did not influence the average latency to find the food reward during acquisition [“treatment” F (1,49) = 3.1, *p* = 0.09; no “treatment” interactions]. We explored these data further with two-way ANOVA split by “genotype”, which showed longer latencies in VEH-treated *APPxPS1* mice than CBD-treated *APPxPS1* mice [“treatment” F (1,24) = 5.1, *p* = 0.03] but not in WT mice [F (1,25) = 0.1, *p* = 0.9] (Figures 2A,B). Follow-up analyses split by “treatment” in WT mice revealed no further significant differences (all *p*-values > 0.1).

Similarly, examination of intermediate-term memory revealed that *APPxPS1* mice had longer latencies than WT mice [“genotype” F (1,49) = 8.0, *p* = 0.007], which was more prominent earlier in acquisition [“days” × “genotype” F (6,294) = 3.2, *p* = 0.004]. Overall, CBD had no effect on intermediate-term memory [“treatment” [F (1,49) = 2.8, *p* = 0.1; no “treatment” interactions]. Split by “genotype,” CBD reduced intermediate-term memory latencies specifically in *APPxPS1* mice [“treatment” F (1,24) = 4.6, *p* = 0.04] but not in WT mice [F (1,25) = 0.1, *p* = 0.9] (Figures 2C,D). Follow-up ANOVA split by “days” revealed no further significant differences (all *p*-values > 0.1). Long-term memory was not different between the genotypes or treatment groups (all “genotype” or “treatment” main effects and interaction *p*-values > 0.05, Supplementary Figures S1A,B).

The speed of mice was also assessed. *APPxPS1* mice were slower than WT controls across days [“days” × “genotype” F (6,294) = 2.6, *p* = 0.02], and CBD treatment affected speed as well [“days” × “treatment” F (6,294) = 3.2, *p* = 0.005] (Figures 2E,F). Split by “genotype,” in *APPxPS1* mice, there was a “days” × “treatment” interaction [F (6,144) = 3.4, *p* = 0.003], suggesting *APPxPS1* VEH mice were slower than CBD-treated *APPxPS1* mice in the first half of acquisition, but *APPxPS1* VEH mice were faster than *APPxPS1* CBD mice by the end of training (Figure 2F). We split by “day” and confirmed “treatment” effects on day 1 only (*p* = 0.02). Similarly, split by “treatment”, VEH-treated *APPxPS1* mice were initially slower than VEH-treated WT mice, but this reached WT levels by mid-training [“genotype” F (1,27) = 5.6, *p* = 0.03; “days” × “genotype” F (6,162) = 2.6, *p* = 0.02]. Splitting by “day” confirmed “genotype” differences on days 1–3 (*p*-values < 0.02). This speed difference was not evident in CBD-treated *APPxPS1* mice (no “genotype” or “days” × “genotype” interaction, all *p*-values > 0.2). *APPxPS1* VEH mice were slower than WT VEH or *APPxPS1* CBD mice only on days 1–3 of acquisition (Figures 2E,F). No other significant differences were detected.

The distance travelled during acquisition is presented in the Supplementary Results section (see also Supplementary Figure S1).

#### 3.2.2 Probe

**FIGURE 3:**
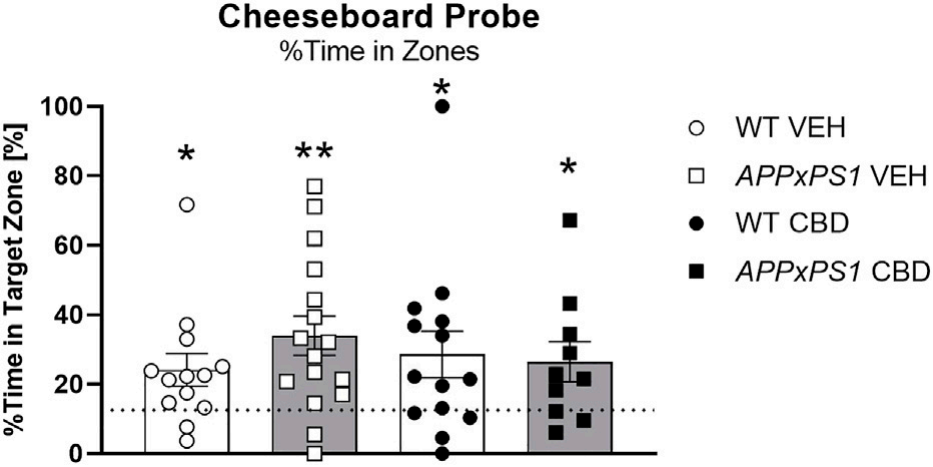
No effect of long-term CBD on recall of spatial memory. Percentage [%] of time spent in the target zone at A probe test in *APPxPS1* and WT mice treated daily with 20 mg/kg CBD for 8 months. Data were analysed using a single sample t-test against chance levels, i.e., 12.5%, corresponding to 1/8 zones. Data presented as mean ± SEM. Significant t-tests against chance are indicated by asterisks (*p < 0.05; **p < 0.01). N = 13 WT VEH, 16 *APPxPS1* VEH, 14 WT CBD, and 10 *APPxPS1* CBD. Abbreviations: *APPxPS1*: *amyloid precursor protein x presenilin 1*; CBD: cannabidiol; VEH: vehicle; WT: wildtype-like.

At probe, all groups spent more time in the target zone than by chance [WT VEH: t = 2.7, df = 12, *p* = 0.03; *APPxPS1* VEH: t = 3.8, df = 15, *p* = 0.002; WT CBD: t = 2.4, df = 13, *p* = 0.03; *APPxPS1* CBD: t = 2.4, df = 9, *p* = 0.04] (Figure 3A).

Data for reversal learning and reversal probe are presented in the Supplementary Results section (see also Supplementary Figures S2–S4).

### 3.3 EPM

**FIGURE 4:**
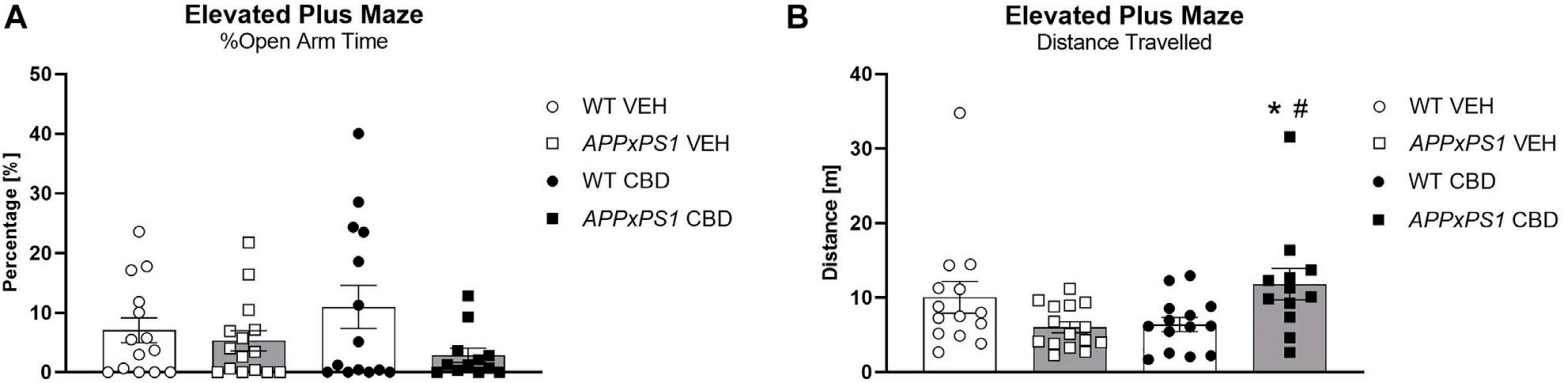
Increased locomotion but unaltered anxiety-like behaviour in *APPxPS1* mice in the elevated plus maze test, following long-term CBD. **(A)** Distance travelled [m] and **(B)** percentage of open arm time [%] in the elevated plus maze test in *APPxPS1* and WT mice treated daily with 20 mg/kg CBD for 8 months. A main effect of “genotype” (*p* = 0.04) was detected in **(A)**. A “genotype” x “treatment” interaction (*p* = 0.004) was detected in **(B)**. Data were analysed using two-way RM ANOVA and presented as mean ± SEM. When data were split by the corresponding factor, significant “genotype” effects in CBD-treated mice are indicated by asterisks (*p < 0.05, vs. WT CBD), and significant “treatment” effects in *APPxPS1* mice are indicated by hash symbols (^#^ *p* < 0.05, vs. *APPxPS1* VEH). N = 14 WT VEH, 15 *APPxPS1* VEH, 14 WT CBD, and 12 *APPxPS1* CBD. Abbreviations: *APPxPS1*: *amyloid precursor protein x presenilin 1*; CBD: cannabidiol; VEH: vehicle; WT: wildtype-like.

*APPxPS1* mice showed more anxiety-like behaviour in the EPM, evidenced by a reduced percentage of time spent in the open arms [“genotype” F (1,51) = 4.3, *p* = 0.04] (Figure 4A). “CBD treatment” did not affect the percentage of open arm time [“treatment” F (1,51) = 0.09, *p* = 0.8; no interaction]. Open arm entries and open arm distance ratios were unaffected by the “genotype” or “treatment” (all main effect and interaction *p*-values > 0.05; Table 2). Although there was no overall effect of the “genotype” or “treatment” on the total distance travelled in the EPM, a “genotype” x “treatment” interaction [F (1,51) = 9.2, *p* = 0.004] indicates chronic CBD increased locomotion in *APPxPS1* mice but not in WT mice (Figure 4B). This was confirmed when data were split by the “genotype”: CBD increased locomotion in *APPxPS1* mice [“treatment” F (1,25) = 7.9, *p* = 0.009] but not WT mice [“treatment” F (1.26) = 2.4, *p* =0 .1]. Also, when data were split by “treatment,” there was a main effect of the “genotype” in CBD-treated mice [F (1,24) = 6.1, *p* = 0.02] but not VEH-treated mice [F (1,27) = 3.4, *p* = 0.08], suggesting greater distance travelled in CBD-treated *APPxPS1* mice than CBD-treated WT mice (Figure 4B).

### 3.4 NORT

The NORT data are presented in Supplementary Figure S5 as WT VEH-treated mice did not demonstrate novel object recognition (i.e. > 50% time *nosing* the novel object) [see a similar lack of object preference: (Kreilaus et al., 2019)] despite this protocol producing significant object novelty recognition in female *APPxPS1* mice previously in our laboratory (Cheng et al., 2014b).

## 4 Discussion

In the current study, we found that long-term preventative oral CBD improved spatial memory acquisition, which was accompanied by changes to speed and locomotion in female *APPxPS1* mice. No effects of CBD treatment were detected on reversal learning or the recall of previously rewarded locations in AD transgenic mice. CBD reduced *freezing*, following the presentation of a discrete cue associated with footshock in both genotypes. Long-term CBD increased the distance travelled in the EPM in *APPxPS1* females but did not affect anxiety-like behaviours in either genotype.

In the CB task, CBD improved the spatial learning of AD transgenic females. *APPxPS1* VEH mice had longer average and intermediate-term memory latencies to find the reward location than *APPxPS1* CBD mice. This effect was not evident in WT mice, suggesting CBD improved spatial learning specifically in AD-affected *APPxPS1* mice but not at baseline (i.e., WT mice), potentially aligning with its low side effect profile (Gaston and Szaflarski, 2018). Interestingly, CBD increased speed and distance travelled by *APPxPS1* mice in the early phases of CB learning (i.e., days 1–3), suggesting effects of CBD on spatial task acquisition may be linked to improved motor function. However, improved locomotion cannot account for all the spatial learning effects observed, as by the end of acquisition *APPxPS1* VEH mice had similar speed yet still slower latencies than *APPxPS1* CBD mice, suggesting *APPxPS1* CBD mice moved more directly to the rewarded location rather than simply moving faster. Strengthening this argument, slower reversal latencies in *APPxPS1* mice also did not correspond with slower speed.

The effects of CBD on motor function require further clarification as the CB and EPM are not traditionally utilised as primary measures for locomotor ability. There are currently no reports of improved locomotor activity by chronic CBD in mouse models of dementia (Cheng et al., 2014a, Cheng et al., 2014c, Coles et al., 2020, de Paula Faria et al., 2022; Khodadadi et al., 2021; Kreilaus et al., 2022; Watt et al., 2020b), and indeed, inconsistent effects of acute and chronic CBD on locomotor activity across a variety of neurological models have been found (reviewed in Calapai et al., 2022). Interestingly, locomotor impairment can occur in some individuals with AD (Scarmeas et al., 2004) and may be linked to *PS1* mutations (Ryan et al., 2016), which may explain some of the locomotor changes observed here in *APPxPS1* mice.

Despite improvements in spatial learning, CBD had no effect on the recall of spatial learning at probe or reversal probe. This reflects previous reports where chronic CBD did not affect spatial memory recall in the CB (Coles et al., 2020; Watt et al., 2020a). We also found no effect of CBD on reversal learning, suggesting oral preventive CBD may not improve performance once the task has been learnt and suggesting only specific cognitive domains may be ameliorated by preventative oral CBD.

The finding of improved spatial learning by CBD is similar to other reports investigating remedial CBD treatment in AD mouse models (i.e., treatment started after spatial learning deficits were present; Amini and Abdolmaleki, 2022; Coles et al., 2020; Martin-Moreno et al., 2011; Watt et al., 2020a). Importantly, ours is the first study to show that long-term CBD can *prevent* the development of some spatial learning deficits in female AD transgenic mice, suggesting CBD may have the potential to *prevent* cognitive impairment in both men (Cheng et al., 2014c) and women. Considering a preventative approach may limit the development or severity of AD pathology and symptoms, our results demonstrate some utility of preventive CBD, although the moderate nature of our findings suggests that preventive CBD may not be as effective as remedial CBD (see Amini and Abdolmaleki, 2022; Coles et al., 2020; Cheng et al., 2014a; Martin-Moreno et al., 2011; Watt et al., 2020a). It is also possible that a higher preventive oral CBD dose may have resulted in more pronounced effects on spatial learning. Nonetheless, by using an oral route of CBD administration in this study and previous work (Cheng et al., 2014c), we provide data which are highly clinically relevant as oral administration is clinically preferable to intravenous or intramuscular injections, and using an oral route significantly boosts the translational power of our findings.

Long-term oral CBD treatment reduced *freezing* in the cue test of all females, regardless of the genotype. Although it is well established that acute systemic CBD can impair fear memory consolidation (Han et al., 2022; Shallcross et al., 2019; review: Stern et al., 2018), including in female mice (Montoya et al., 2020), effects of chronic CBD on fear memory have had limited investigation, and chronic CBD does not affect fear memory acquisition (Cheng et al., 2014a; 2014c). Considering CBD-induced differences in *freezing* were very limited in this study, future research should consider evaluating the effects of long-term CBD on fear learning in female mice.

Chronic CBD had no effect on anxiety-like behaviours in the EPM, and this corresponds with previous reports. Although the anxiolytic-like effects of acute systemic CBD are well established [reviews: (Blessing et al., 2015; Wright et al., 2020)], the anxiolytic-like effects of chronic CBD are less clear. Chronic low-dose CBD (up to 30 mg/kg) does not affect anxiety-like behaviour in the EPM in *APPxPS1* male mice (Cheng et al., 2014a; Cheng et al., 2014c) or in outbred rats and mice (Schiavon et al., 2016; Murphy et al., 2017; Gall et al., 2020). However, high-dose chronic CBD (30-100 mg/kg i. p. or subcutaneous, s. c.) can decrease anxiety-like behaviour in the EPM in mice (Long et al., 2012; Fogaca et al., 2018; Patra et al., 2020). It is possible that higher doses of CBD are necessary for anxiolytic-like effects, following long-term administration. Also, most studies use systemic injections (i.p. or s.c.) to evaluate the anxiolytic effects of CBD (Long et al., 2012; Schiavon et al., 2016; Murphy et al., 2017; Fogaca et al., 2018; Gall et al., 2020; Patra et al., 2020), and it is unknown if the oral route may alter CBD’s effects on anxiety-like behaviour.

The mechanisms by which CBD exerts pro-cognitive effects are poorly understood, but recent reports suggest potential mechanisms. Chronic CBD can enhance the immune response and increase hippocampal autophagy in *APPxPS1* mice (Hao and Feng, 2021). An enhanced immune response by CBD may also drive increased microglial migration and reduced nitrite generation (Martin-Moreno et al., 2011), which can facilitate Aβ phagocytosis and decrease hippocampal Aβ plaque load, thus improving cognition in *APPxPS1* mice (Watt et al., 2020a; Hao and Feng, 2021). Alternatively, it is possible that CBD ameliorates hippocampal synaptic plasticity deficits to improve spatial learning as CBD pretreatment prevents Aβ_1–42_-mediated LTP deficits in mouse hippocampal slices (Hughes and Herron, 2019). Examining the brain pathology in these mice to determine the mechanism/s of CBD in this instance would be a valuable focus for future research studies.

It is possible there are sex differences in the effects of CBD on cognition in *APPxPS1* mice. In the present study, long-term CBD reversed spatial learning impairment in female *APPxPS1* mice, while in male *APPxPS1* mice, long-term CBD reversed social recognition impairment (Cheng et al., 2014c). It should be noted that male and female *APPxPS1* mice show deficits in different cognitive behavioural domains (Cheng et al., 2013; Cheng et al., 2014b; Richetin et al., 2017), and this is why the behavioural tests conducted in the present study were not identical to those conducted in male *APPxPS1* mice treated with long-term oral CBD (Cheng et al., 2014c). Nonetheless, it is possible that CBD could have sex-specific effects on cognition, and this may be related to sex-specific differences in hippocampal dendritic spine density. Hippocampal dendritic spine density is reduced in female *APPxPS1* mice compared to WT female mice, where this effect is not as pronounced in male *APPxPS1* mice (Richetin et al., 2017). Dendritic spine density is associated with spatial memory function (Mahmmoud et al., 2015), and CBD can ameliorate stress-induced reductions in the hippocampal spine density in mice (Fogaca et al., 2018). Thus, in female *APPxPS1* mice, CBD may increase the hippocampal dendritic spine density to improve spatial memory.

A final consideration for the current study is that of the administration route. This study and others (Cheng et al., 2014c) gave 20 mg/kg CBD orally, whereas other work has administered 20 mg/kg CBD i. p. (Cheng et al., 2014a). In mice, i. p. administration leads to a faster peak brain concentration of CBD than oral administration (Deiana et al., 2012), and the plasma concentration of i. v. CBD is consistently higher than oral CBD for up to 24 h post-administration (Xu et al., 2019). The bioavailability of i. v. or i. p. CBD is close to 100% (Zgair et al., 2016; Xu et al., 2019), whereas oral CBD is 8.6% (Xu et al., 2019). This suggests a faster and more potent effect of i. p. CBD than oral CBD even at the same CBD dose, which may explain why the effects of oral CBD are not as pronounced as for i. p. CBD, e.g., i. p. CBD reversed both object and social memory impairment in male *APPxPS1* mice (Cheng et al., 2014a), but oral CBD only reversed social memory impairment in male mice (Cheng et al., 2014c).

In conclusion, we found moderate effects of long-term oral CBD treatment on the acquisition of spatial learning by CBD in a female mouse model of familial AD. This suggests that *preventive* CBD may help limit some cognitive impairment in women with AD.

## Data Availability

The original contributions presented in the study are included in the article/Supplementary Material; further inquiries can be directed to the corresponding author.
