# Supplementary material for: Effect of long-term cannabidiol on learning and anxiety in a female Alzheimer’s disease mouse model
Source: Front Pharmacol. 2022 Sep 27;13:931384. doi: 10.3389/fphar.2022.931384 (PMC9551202; doi:10.3389/fphar.2022.931384)
Supplement: Supplementary file 1 [file Table1.DOCX]

Supplementary Material

**Supplementary Results: Cheeseboard**

Distance travelled during acquisition: Analysis of distance travelled during acquisition revealed a ‘days’ x ‘genotype’ x ‘treatment’ interaction [F(6,294) = 2.2, *p* = .047; Supp Fig. 1C-D]. Split by ‘genotype’, a ‘days’ x ‘treatment’ interaction in *APPxPS1* mice suggests VEH *APPxPS1* mice travelled less than *APPxPS1* CBD mice early in training [F(6,144) = 3.2, *p* = .006; no ‘treatment’ effect]. This was confirmed when we split by ‘days’ and found reduced distance in VEH *APPxPS1* vs CBD *APPxPS1* on day 1 only (*p* = .02). There were no treatment effects or interactions in WT mice (all *p*’s > .1; Supp Fig. 1C-D). Similarly, split by ‘treatment’, VEH-treated *APPxPS1* mice travelled less than corresponding WT mice across days [‘days’ x ‘genotype’ interaction: F(6,162) = 5.2, *p* < .0001]. This genotype difference was only apparent early in testing, as when split by ‘days’, a ‘genotype’ effect was only evident on day 1 (*p* < .001; Supp Fig. 1C). There were no ‘genotype’ or ‘days’ x ‘genotype’ interactions in CBD-treated mice (all *p*’s > .5; Supp Fig. 1D). This suggests *APPxPS1* VEH mice travelled less than WT VEH and *APPxPS1* CBD on day 1 of acquisition.

Reversal: Assessing latency to find the food reward during reversal, all mice learned to find the food reward [‘days’ F(3,147) = 55.3, *p* < .0001] but a trend for a ‘days’ x ‘genotype’ interaction [F(3,147) = 2.5, *p* = .06; Supp Fig 2A-B] suggests that latencies to find the reward across days tended to be greater in *APPxPS1* mice. CBD treatment did not affect reversal learning [‘treatment’ F(1,49) = 1.5, *p* = .2; no ‘treatment’ interactions with ‘days’ or ‘genotype’ or both].

Intermediate-term memory during reversal was affected by both genotype and treatment [‘days’ x ‘genotype’ x ‘treatment’ interaction F(3,147) = 5.4, *p* = .002]. Importantly, when split by ‘genotype’, a ‘days’ x ‘treatment’ interaction was only present in *APPxPS1* mice [F(3,72) = 4.6, *p* = .005], suggesting shorter latencies in *APPxPS1* CBD mice on the first day of reversal compared to *APPxPS1* VEH mice (Supp Fig 2C-D); but we found no further ‘treatment’ differences when we split by ‘days’ (all *p*’s > .05). Split by ‘treatment’, *APPxPS1* VEH mice initially had longer latencies than WT VEH mice but this reached WT VEH levels as training progressed [as indicated by a ‘days’ x’ ‘genotype’ interaction: F(3,81) = 4.1, *p* = .009]. This was confirmed when we split by ‘days’ as ‘genotype’ differences were found on days 1 (*p* = .009) and 2 (*p* = .01). *APPxPS1* CBD mice showed similar latencies to WT CBD mice on day 1 of reversal, but higher latencies thereafter [‘days’ x ‘genotype’ F(3,81) = 4.1, *p* = .009] (Supp Fig 2C-D). Split by ‘days’, we confirmed higher latencies in *APPxPS1* CBD than WT CBD mice on days 2 and 3 of reversal (both *p*’s = .03).

Long-term memory during reversal was not different between the genotypes or treatment groups (all ‘genotype’ or ‘treatment’ main effects and interaction *p*’s > .05, Supp Fig. 3A-B).

*APPxPS1* mice were not slower than WT controls in the first trial of reversal training [‘genotype’ F(1,49) = .2, *p* = .7], and speed was not different between the genotypes across all trials and all days of reversal learning [‘genotype’ F(1,49) = .2, *p* = .7; no interactions] (Supp Fig 2E-F).

Distance travelled during reversal testing was modified by genotype and treatment [‘days’ x ‘genotype’ F(3,147) = 2.8, *p* = .04; ‘days’ x ‘treatment’ F(3,147) = 3.0, *p* = .03]. Split by ‘genotype’, *APPxPS1* VEH mice appeared to travel further than *APPxPS1* CBD mice early in reversal only [‘days’ x ‘treatment’ F(3,72) = 2.9, *p* = .04] (Supp Fig. 3C-D) but this was not clarified further when split by ‘days’ (all *p*’s > .05). There were no ‘treatment’ effects or interactions in WT mice (all *p*’s > .2). Split by ‘treatment’, CBD-treated *APPxPS1* mice had greater distance travelled than WT CBD mice on some reversal days (Supp Fig. 3D) [‘days’ x ‘genotype’ F(3,66) = 3.1, *p* = .04; no other genotype effects or interactions]; but when split by ‘days’ we did not identify genotype effects on specific reversal days (all *p*’s > .1). There were no ‘genotype’ effects or interactions in VEH-treated mice (all *p*’s > .2).

Reversal Probe: At Reversal Probe, all groups spent more time in the target zone than chance [WT VEH: t = 3.3, df = 12, *p* = .006; *APPxPS1* VEH: t = 2.5, df = 15, *p* = .03; WT CBD: t = 3.2, df = 13, *p* = .007; *APPxPS1* CBD: t = 3.1, df = 9, *p* = .01] (Supp Fig 4).

**
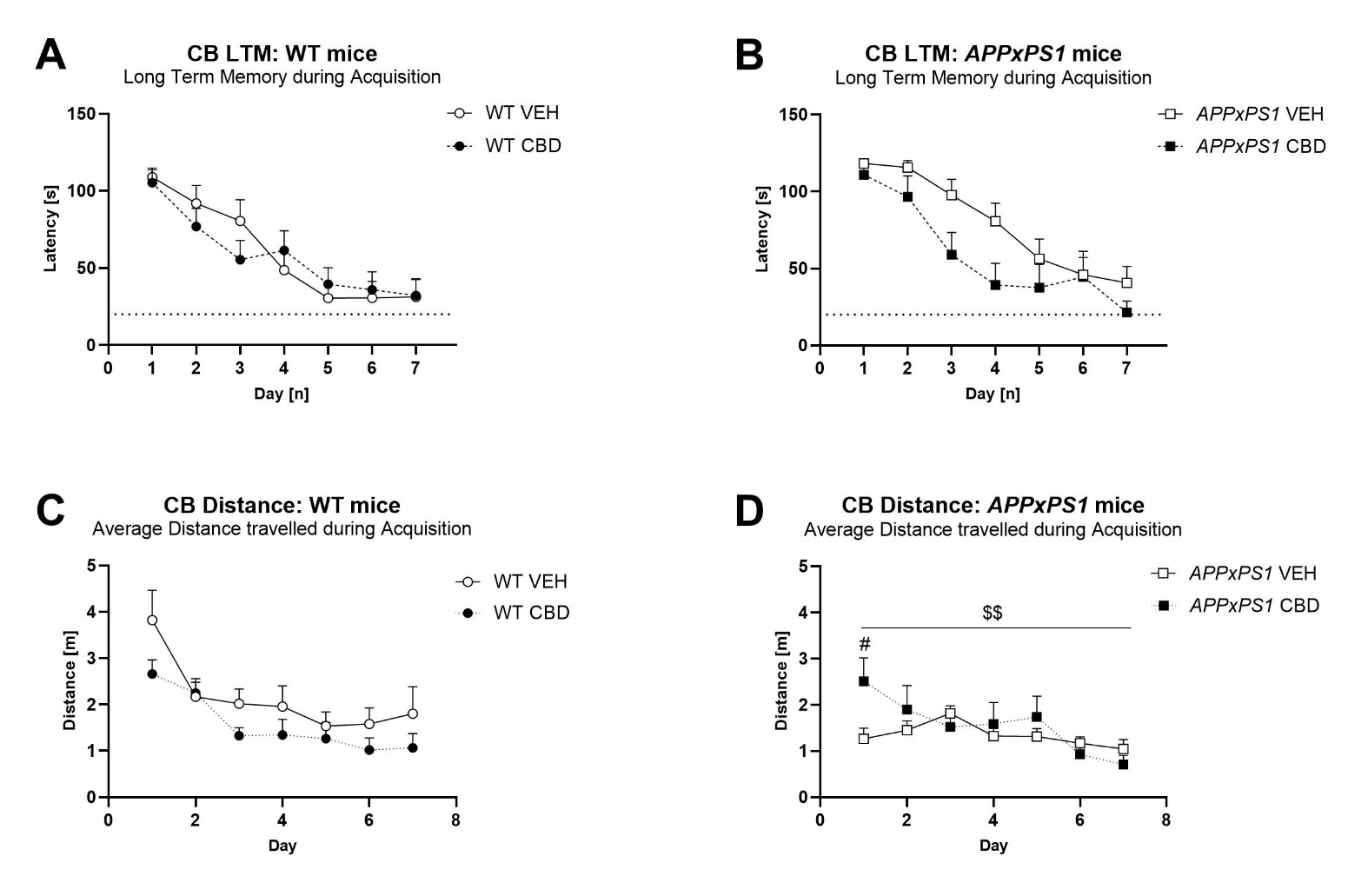
**

**Supplementary Figure 1.** **Long-term memory and distance travelled during cheeseboard acquisition in WT and *APPxPS1* mice.** Latency [s] to find the food reward in the first trial of each day i.e. long-term memory is presented for A-B) acquisition in WT and *APPxPS1* mice treated daily with 20 mg/kg CBD for 8 months. The dotted line indicates the 20 s cutoff threshold. Distance travelled [m] to find the food reward is presented in C-D). In C-D) there was a ‘days’ x ‘genotype’ x ‘treatment’ interaction (*p* = .047). When split by ‘treatment’, there was a ‘days’ x ‘genotype’ interaction in VEH-treated mice (*p* < .0001). Data analysed using three-way RM ANOVA and presented as means ± SEM. When data were split by corresponding factor, significant effects of ‘treatment’ on individual days are indicated by hash symbols (^#^*p* < .05), significant interactions between ‘days’ and ‘treatment’ are indicated by ‘$’ (^$$^*p* < .01). N = 14 WT VEH, 16 *APPxPS1* VEH, 14 WT CBD, 13 *APPxPS1* CBD. Abbreviations: *APPxPS1*: *amyloid precursor protein x presenilin 1*; CB: cheeseboard; CBD: cannabidiol; LTM: long-term memory; VEH: vehicle; WT: wild type-like.


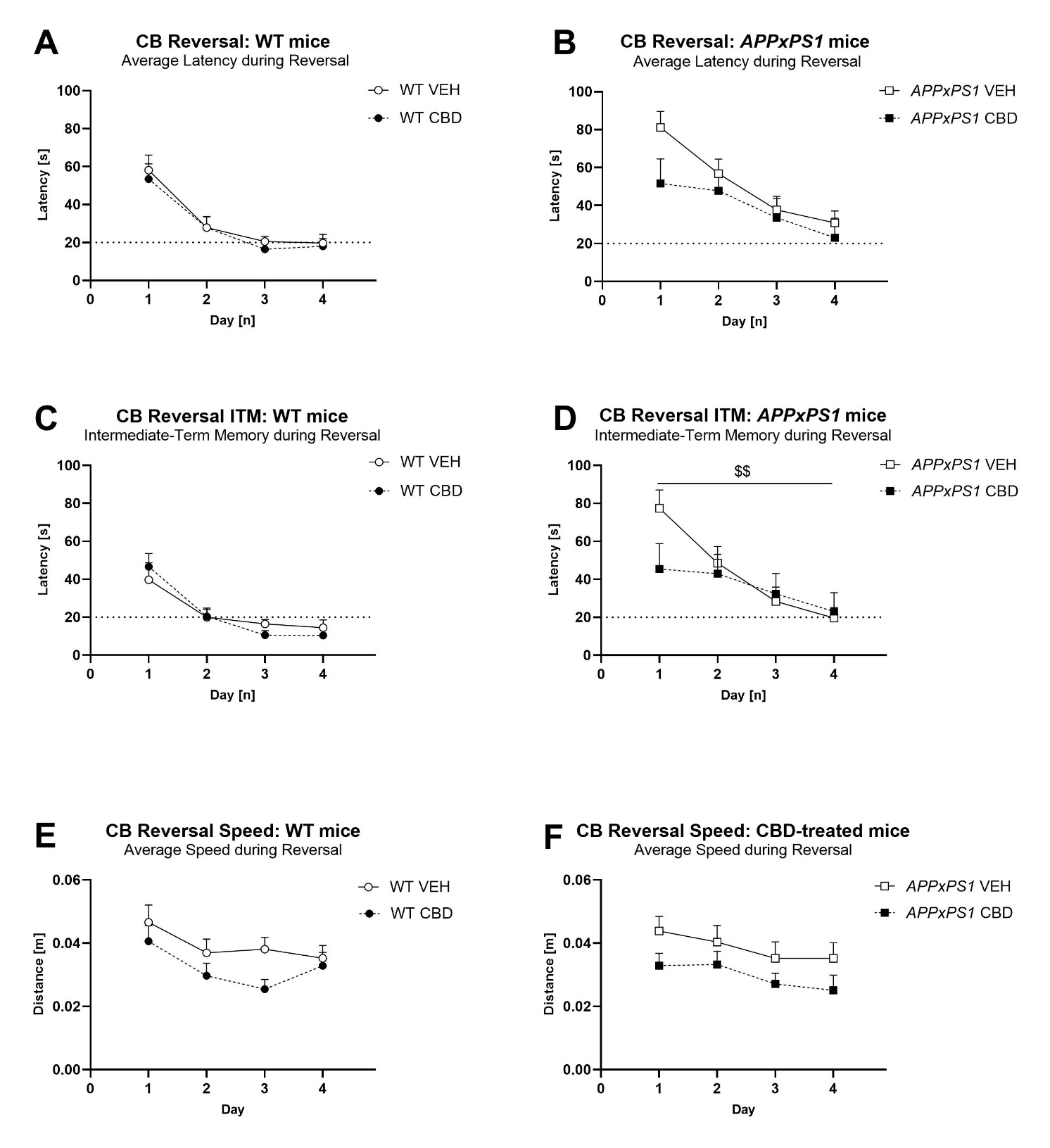


**Supplementary Figure 2. Limited effects of long-term CBD on reversal learning in WT and *APPxPS1* mice.** Latency [s] and speed [m/s] when finding the food reward during cheeseboard reversal in WT and *APPxPS1* mice treated daily with 20 mg/kg CBD for 8 months. A-B) Average latency [s] to find the food reward (averaged across 3 trials per day) during cheeseboard reversal learning. C-D) Intermediate term memory latency [s] (i.e. average latency for trials 2 and 3 of each day) during reversal. E-F) Average speed [m/s] (averaged across 3 trials per day) during cheeseboard reversal. The dotted line in A-D) indicates the 20 s cutoff threshold for reversal. In C-D), a ‘days’ x ‘genotype’ x ‘treatment’ interaction (*p* = .002) was detected, and when split by ‘treatment’, we found a ‘genotype’ x ‘days’ interaction in VEH-treated mice (*p* = .009). Data analysed using three-way RM ANOVA and presented as means ± SEM in separate graphs for visual clarity. When data were split by corresponding factor, interactions between ‘days’ and ‘treatment’ are indicated by ‘$ (^$$^*p* < .01). N = 13 WT VEH, 16 *APPxPS1* VEH, 14 WT CBD, 10 *APPxPS1* CBD. Abbreviations: *APPxPS1*: *amyloid precursor protein x presenilin 1*; CBD: cannabidiol; VEH: vehicle; WT: wildtype-like.


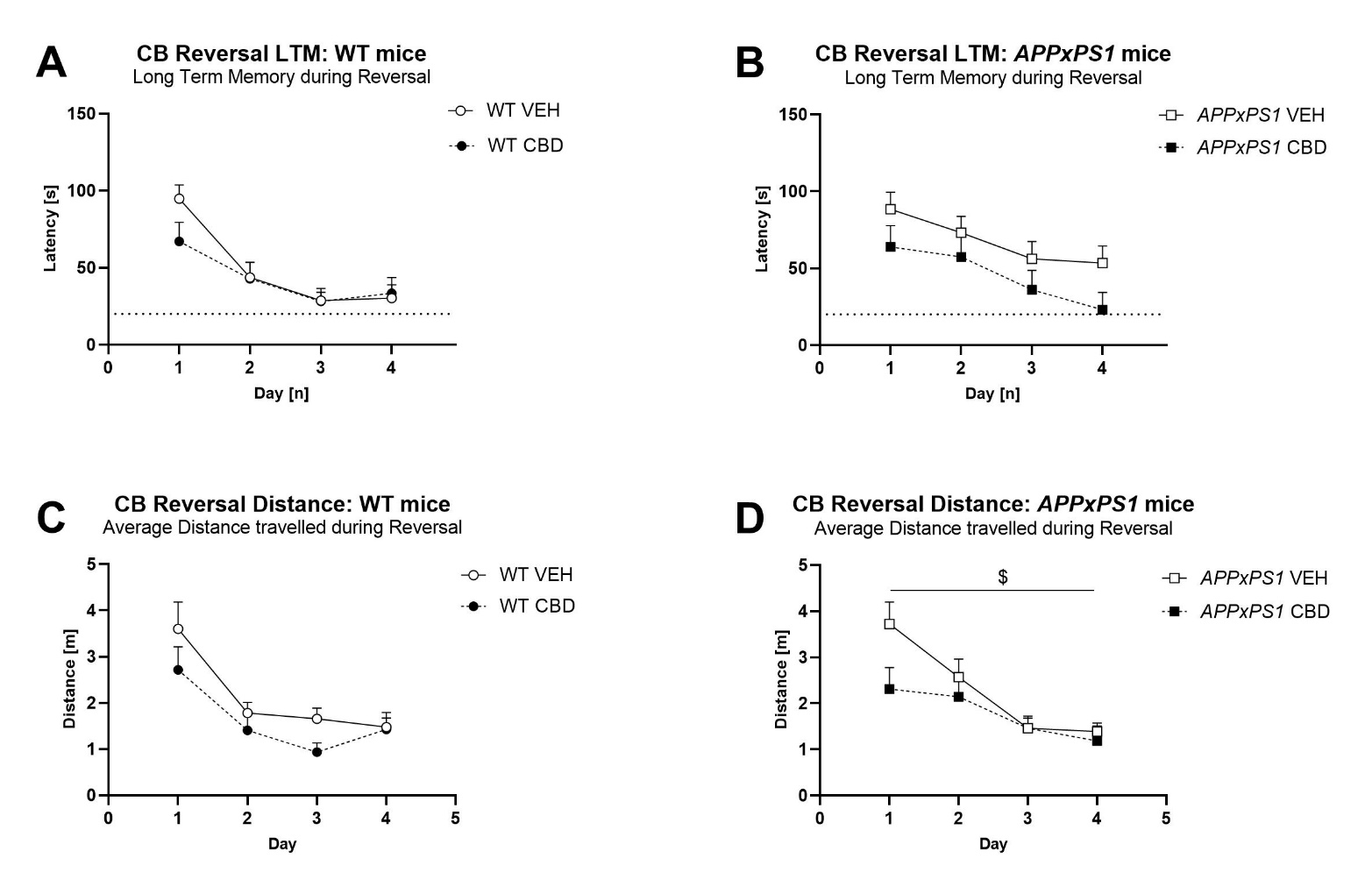


**Supplementary Figure 3.** **Long-term memory and distance travelled during cheeseboard reversal in WT and *APPxPS1* mice.** Latency [s] to find the food reward in the first trial of each day i.e. long-term memory is presented in A-B) for WT and *APPxPS1* mice treated daily with 20 mg/kg CBD for 8 months. The dotted line indicates the 20 s cutoff threshold. Distance travelled [m] during reversal is presented for C-D). In C-D), there was a ‘days’ x ‘genotype’ (*p* = .04) and a ‘days’ x ‘treatment’ interaction (*p* = .03). Split by ‘genotype’, there was a ‘days’ x ‘treatment’ interaction in CBD-treated mice (^$^*p* = .04). N = 1w4 WT VEH, 16 *APPxPS1* VEH, 14 WT CBD, 13 *APPxPS1* CBD. Abbreviations: *APPxPS1*: *amyloid precursor protein x presenilin 1*; CB: cheeseboard; CBD: cannabidiol; LTM: long-term memory; VEH: vehicle; WT: wild type-like.


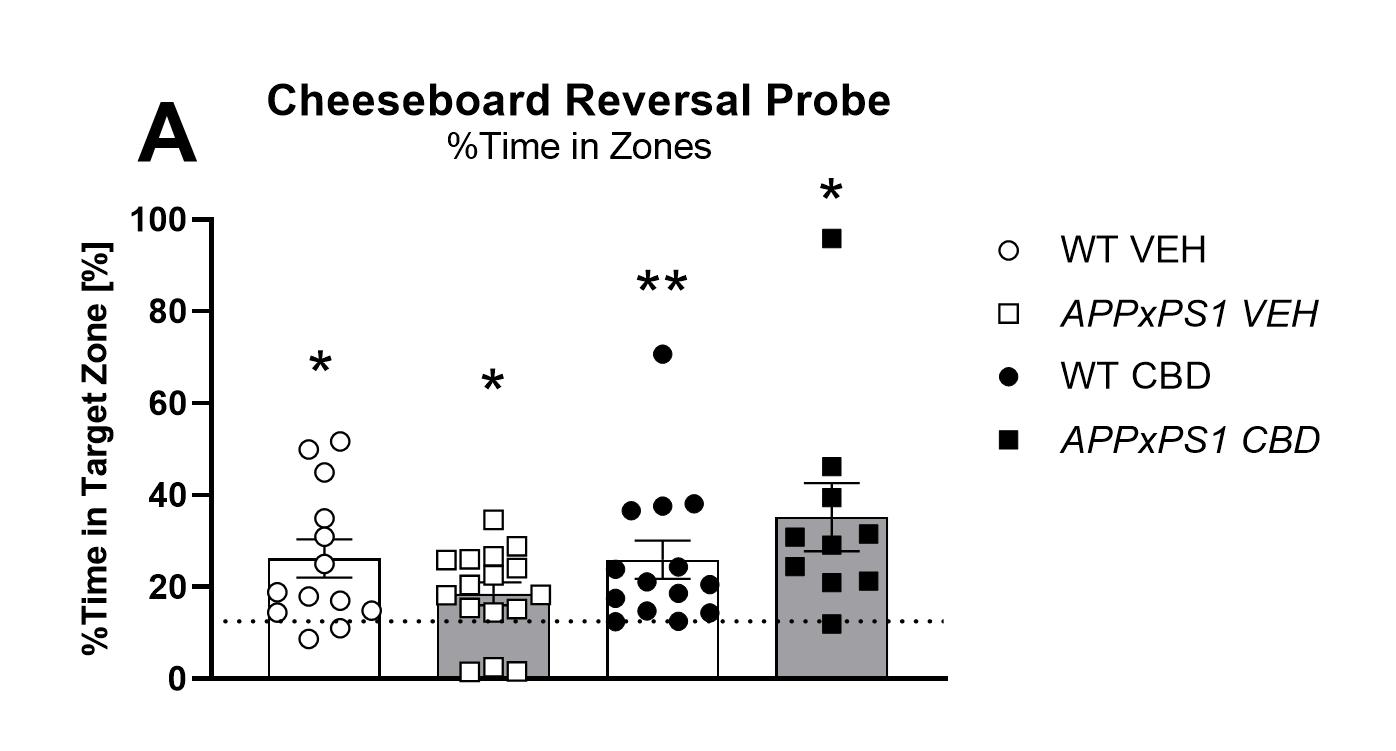


**Supplementary Figure 4. No effect of *APPxPS1* genotype or long-term CBD on recall of spatial memory.** Percentage [%] of time spent in the target zone at A) Recall Probe test in WT and *APPxPS1* mice treated daily with 20 mg/kg CBD for 8 months. Data analysed using single sample t-test against chance levels i.e. 12.5%, corresponding to 1/8 zones. Data presented as means ± SEM. Significant t-tests against chance are indicated by asterisks (**p* < .05, ***p* < .01). N = 13 WT VEH, 16 *APPxPS1* VEH, 14 WT CBD, 10 *APPxPS1* CBD. Abbreviations: *APPxPS1*: *amyloid precursor protein x presenilin 1*; CBD: cannabidiol; VEH: vehicle; WT: wildtype-like.


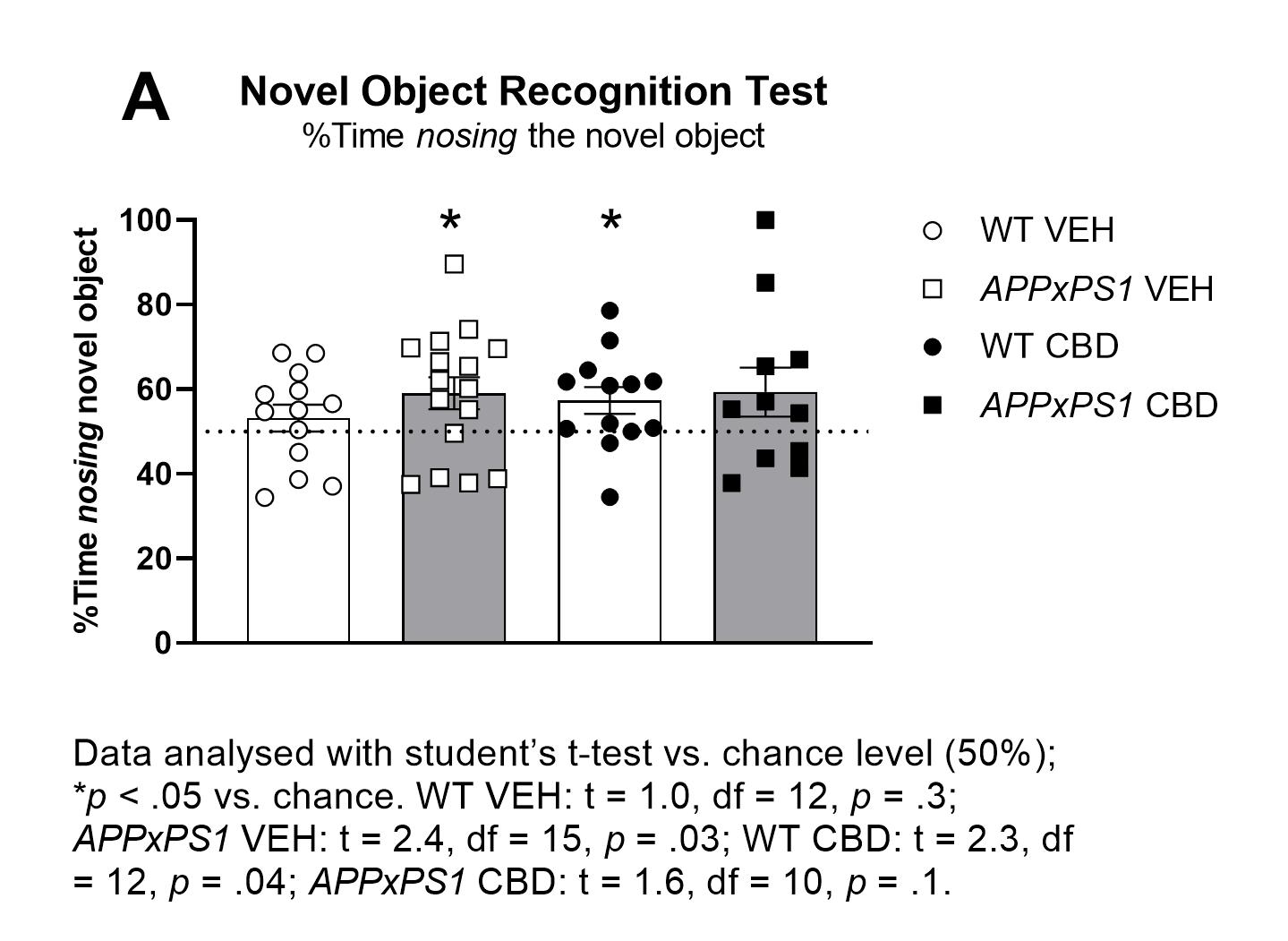


**Supplementary Figure 5. Novel object recognition in WT and *APPxPS1* mice.** Percent time [%] *nosing* the novel object (compared to the familiar object) in WT and *APPxPS1* mice. Data analysed with student’s t-test vs. chance level (50 %); **p* < .05 vs. chance. WT VEH: t = 1.0, df = 12, *p* = .3; *APPxPS1* VEH: t = 2.4, df = 15, *p* = .03; WT CBD: t = 2.3, df = 12, *p* = .04; *APPxPS1* CBD: t = 1.6, df = 10, *p* = .1. N = 13 WT VEH, 16 *APPxPS1* VEH, 13 WT CBD, 11 *APPxPS1* CBD. Abbreviations: *APPxPS1*: *amyloid precursor protein x presenilin 1*; CBD: cannabidiol; VEH: vehicle; WT: wild type-like.
